# Crosstalk between DNA methylation and gene expression in colorectal cancer, a potential plasma biomarker for tracing this tumor

**DOI:** 10.1038/s41598-020-59690-0

**Published:** 2020-02-18

**Authors:** Mohammad Amin Kerachian, Ali Javadmanesh, Marjan Azghandi, Afsaneh Mojtabanezhad Shariatpanahi, Maryam Yassi, Ehsan Shams Davodly, Amin Talebi, Fatemeh Khadangi, Ghodratollah Soltani, Abdorasool Hayatbakhsh, Kamran Ghaffarzadegan

**Affiliations:** 1 Medical Genetics Research Center, Mashhad University of Medical Sciences, Mashhad, Iran; 2 Department of Medical Genetics, Faculty of Medicine, Mashhad University of Medical Sciences, Mashhad, Iran; 3 Cancer Genetics Research Unit, Reza Radiotherapy and Oncology Center, Mashhad, Iran; 4 Department of Animal Science, Faculty of Agriculture, Ferdowsi University of Mashhad, Mashhad, Iran; 5 Institut Universitaire de Cardiologie et de Pneumologie de Québec, Université Laval, Québec, Québec, Canada; 6 Department of Gastroenterology, Reza Radiotherapy and Oncology Center, Mashhad, Iran; 7 Razavi Cancer Research Center, Razavi Hospital, Imam Reza International University, Mashhad, Iran

**Keywords:** Cancer epigenetics, Cancer epigenetics

## Abstract

Colorectal cancer (CRC), the second leading cause of cancer mortality, constitutes a significant global health burden. An accurate, noninvasive detection method for CRC as complement to colonoscopy could improve the effectiveness of treatment. In the present study, SureSelectXT Methyl-Seq was performed on cancerous and normal colon tissues and *CLDN1*, *INHBA* and *SLC30A10* were found as candidate methylated genes. MethyLight assay was run on formalin-fixed paraffin-embedded (FFPE) and fresh case and control tissues to validate the methylation of the selected gene. The methylation was significantly different (p-values < 2.2e-16) with a sensitivity of 87.17%; at a specificity cut-off of 100% in FFPE tissues. Methylation studies on fresh tissues, indicated a sensitivity of 82.14% and a specificity cut-off of 92% (p-values = 1.163e-07). The biomarker performance was robust since, normal tissues indicated a significant 22.1-fold over-expression of the selected gene as compared to the corresponding CRC tissues (p-value < 2.2e-16) in the FFPE expression assay. In our plasma pilot study, evaluation of the tissue methylation marker in the circulating cell-free DNA, demonstrated that 9 out of 22 CRC samples and 20 out of 20 normal samples were identified correctly. In summary, there is a clinical feasibility that the offered methylated gene could serve as a candidate biomarker for CRC diagnostic purpose, although further exploration of our candidate gene is warranted.

## Introduction

Colorectal cancer (CRC) constitutes a significant global health burden, leading to over 862,000 deaths globally in 2018. It is the second main cause of cancer mortality in the world and currently stands as the third most common cancer, with a yearly incidence of over one million and eight thousand cases worldwide^1^. Its leading cause of death is due to liver metastasis with a median survival rate of approximately 30 months. Generally, half of the patients with CRC develop tumor recurrences^2^. For early-diagnosed CRC patients, the 5-year survival rates are approximately 90% but this lowers to less than 10% in patients with extensive metastases. Thus, the most effective approach to reduce CRC incidence and its mortality is early detection of colonic lesions^3^. Fortunately, because of implementation and growth of wide spread cancer screening assays, such as colonoscopy as well as increasingly effective therapies, the mortality rate of CRC is lowering in many countries^2^.

Flexible colonoscopy is considered as the golden screening option for this type of cancer. This procedure is an invasive method and has the highest rate of complications among other screening methods. Besides, it has about 5% error probability, and is relatively expensive. A large number of people are reluctant to undergo colonoscopy because of the fear of pain, and discomfort or even embarrassment^4^. Altogether, colonoscopy includes patient non-compliance, invasiveness, and uncertain cost-effectiveness, which poses challenges in population-based screening^5^. All other available CRC screening methods seem to be used less frequently for various reasons. An ideal screening tool should be inexpensive, noninvasive, easy to perform, and accurate with high sensitivity for advanced adenomas or early cancer. It should be widely available and specific enough to avoid unnecessary second level tests. Finally, it should reduce mortality rate significantly above the figure of approximately 30–40%. The fecal occult blood test (FOBT), used in population-based screening, was the first and cheapest noninvasive option, which is widely available now. However, this method requires several attempts and has a low sensitivity (59.7%). In recent years, more specific and sensitive methods such as the fecal immunochemical test (FIT) have replaced FOBT by the guidelines for CRC screening panel^4^.

In 2017, Wu *et al*. proposed a miRNA-based approach to quantify fecal blood levels over a broad, clinically relevant range as a new marker class for FOBT. Candidate miRNA markers (hsa-miR-144-3 p, 144-5 p, 451 a, 486-5 p, 363-3 p, 20b-5 p) were identified by small RNA sequencing of human whole blood compared with colorectal epithelia^6^. In addition, gut microbiota changes in the intestinal microbiota composition in CRC patients have also been reported as CRC biomarkers in several studies^7^. In the last two decades, many research concerning CRC potential biomarkers have focused on analyzing the fecal DNA^8^. Stool DNA test was approved by US Food and Drug Administration (FDA) in August 2014 and was available under the commercial name of Cologuard^®^ (Exact Sciences, Madison, WI, USA). It is recommended for the average risk screening in asymptomatic 50–85 years old^9^. Later, in April 2016 Epi proColon was introduced as the first FDA-approved blood-based CRC screening test. Epi proColon 2.0 CE is based on methylated *septin 9* (*SEPT9*) gene from the circulating cell-free DNA (cfDNA) in the blood. It is now accessible in Europe and different nations such as China^10,11^. This test offers improved sensitivity and specificity over the first generation Epi proColon test^11^ although it has not been recommended by the US Preventive Services Task Force in their most recent guidelines due to its very low sensitivity for cancer^12^. Based on a meta-analysis conducted by Song *et al*., the performance of the blood methylated *SEPT9* assay is superior to the serum protein biomarkers and FIT in symptomatic patients, while it seems to be less potent than FIT in asymptomatic patients^13^. In a recent meta-analysis comparing CRC patients with healthy subjects, the pooled sensitivity and specificity of *SEPT9* methylation were 0.74 (95% CI: 0.61–0.84) and 0.96 (95% CI: 0.95–0.97), respectively^14^.

During recent years, the non-protein biomarkers so-called “liquid biopsy” of circulating tumor cells and circulating DNA or exosome products has drawn great attention^4,15^. Developing new biomarkers is relied on understanding the mechanisms exploited by cancer. A variety of biomarkers such as diagnostic, preventive and prognostic has been offered for CRC based the cellular and molecular tumorigenesis.

CRC represents a group of molecularly heterogeneous diseases characterized by genetic and epigenetic alterations such as genetic mutations and DNA methylation, along with a tumorigenesis sequence^2^. DNA methylation alteration is a hallmark of not only CRC, but also virtually all tumor types. It represents a sophisticated molecular mechanism for annotating genetic information^16^, and in most cases, modulates the genetic expression level. Although the mechanism and the role of DNA methylation is not completely understood, it is assumed that DNA methylation could affect the binding of the transcription factors to their DNA target sites and subsequently, alter the expression of downstream genes^17^. In tumor cells, abnormal DNA methylation could be commonly classified into two groups: (1) site specific CpG island promoter hypermethylation, (2) global DNA hypomethylation. Several studies in tumor cells have elucidated site specific CpG island promoter hypermethylation in tumor suppressor genes and global DNA hypomethylation in repetitive sequences^2^. Besides, the gene body DNA hypermethylation in oncogenes is concerned with gene overexpression, suggesting that the genes regulated by DNA methylation are driving elements in tumorigenesis^18^. CpG islands (CGIs), shores and shelves that are the 2 kb and 4 kb regions immediately upstream and downstream of the CGI boundaries respectively, are also subjected to methylation. A recent study has shown that cancer specific differentially methylation occurs more often within CGI shores with low CpG densities, rather than CGIs with high CpG densities, indicating a distinct methylation profile in cancer^19^.

Today, DNA methylation could be mapped by whole-genome bisulfite sequencing (WGBS), which is currently the state-of-the-art technology to achieve a comprehensive, nucleotide-resolution view of the entire epigenome. The methylated differences among samples are generally determined by differentially methylated regions (DMRs). The detection of methylation alterations between cancer patients and normal individuals requires to take the variation of relative methylation within each group into consideration. Such variations could be associated with several technical and biological issues including unequal cytosine conversion rates, different library preparation protocols, different technical methylation assays and the existence of the natural epigenetic variations among individuals^17^.

In this study, we sought to identify candidate genes for the accurate detection of CRC as novel biomarkers based on the methylation profile and gene expression. There is a clinical feasibility that the methylation biomarkers could be used for detection of CRC. An accurate, noninvasive method based on the methylation biomarkers for early detection and screening of CRC as a complement to colonoscopy has the potential to improve patient satisfaction and the overall effectiveness.

## Results

### Discovery

#### Methylation sequencing data processing and quality control

**Figure 1:**
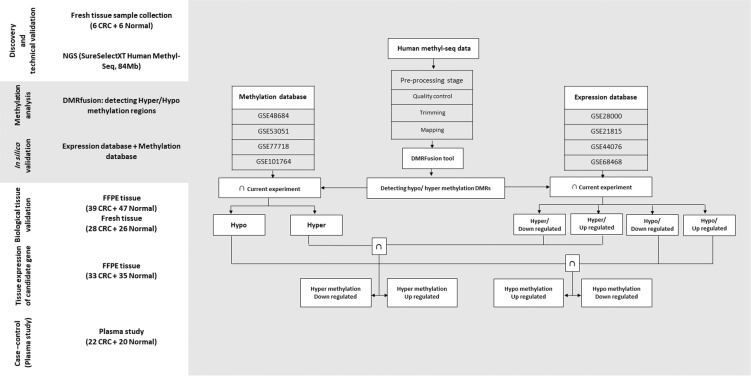
Project workflow. Discovery and technical validation set: CRC and normal DNA samples collected for the study of NGS. Bioinformatic analysis set: Bioinformatic analysis among Methylation and Expression database and current expriment for detecting robust hypo- hyper methylation genes. Biological validation and tissue expression set: CRC and normal FFPE samples used for Biological validation and candidate gene expression by real time PCR. Plasma study sets: CRC and normal plasma samples used for methylation study of candidate gene.

The platform of this study is illustrated in Fig. 1. To perform a genome-wide analysis of DNA methylation in CRC, we applied SureSelect^**XT**^ Human Methyl-Seq approach with 101 read length that generates 57–76 million Illumina sequencing reads for each sample. Of these, 88.5% to 89.8% were successfully mapped to either strand of the human genome (GRCh37/19). The average number of times that each CpG has been sequenced per sample was between 19.26X and 24.43X (Supplementary Table S1).

#### DMR detection

**Figure 2:**
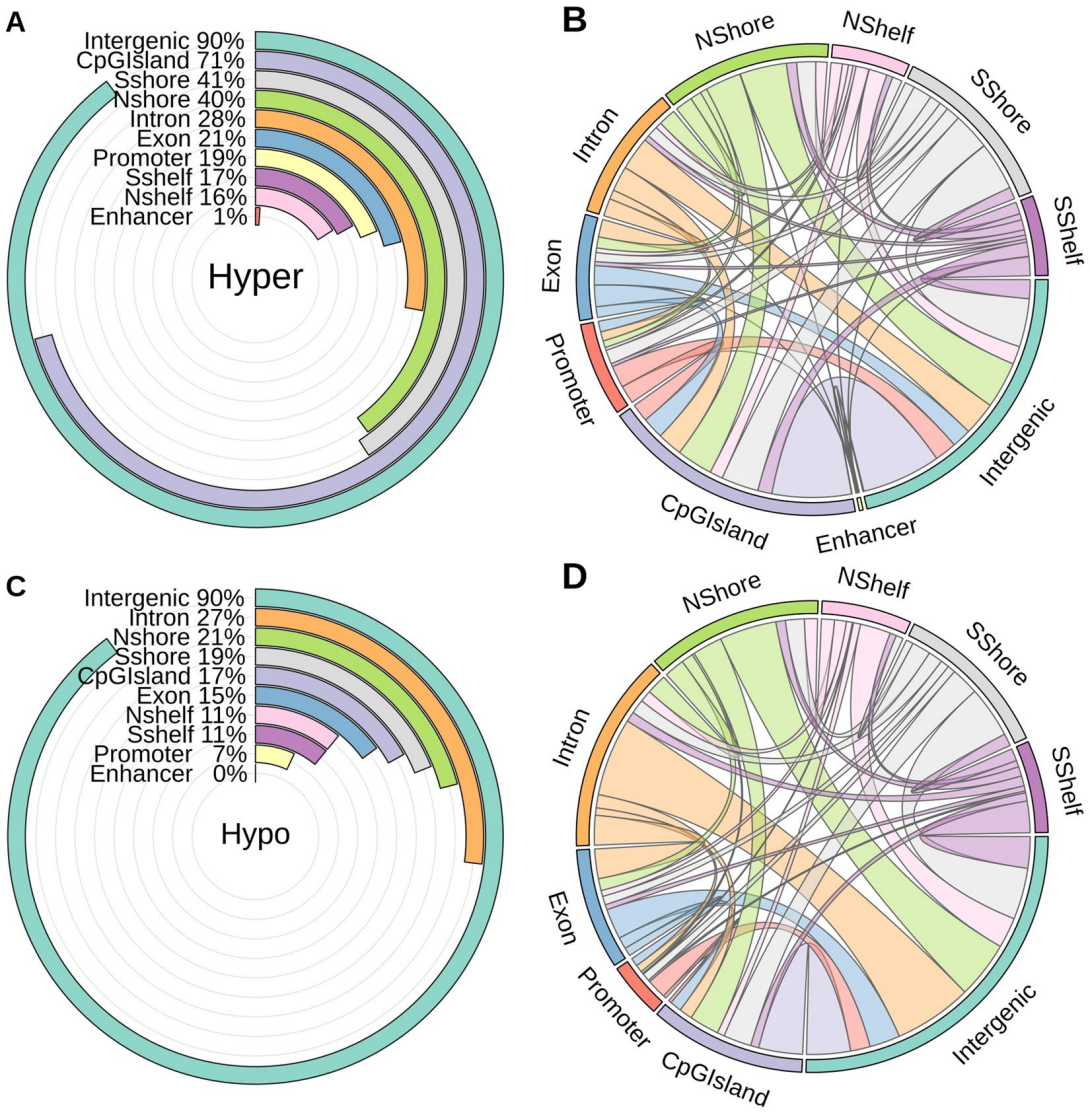
Statistic information of Hyper-Hypo Methylation DMRs annotation.

Several thousand hyper and hypo methylation DMRs were detected in multi-samples by comparing CRC and normal groups. In total, we identified 5780 hyper and 8909 hypo DMRs with the length of more than 200 bp, with the highest fold difference score between these two groups, and also with the p-value and false discovery rate (FDR) less than 0.05. Figure 2A,C indicate that the majority of detected DMRs in both hyper and hypo categories were located in the intergenic regions (90%). Seventy one percent of the hyper DMRs were annotated in CpG islands, in comparison with only 17% of the hypo DMRs allocated in these regions. It is worth noting that, the most percentage of the hyper DMRs regions were located in CGI shores, exons, promoters and CGI shelves.

As the detected DMRs were larger than 200 bp, some of them were expanded in several regions of the genome, possessing more than one annotation feature. Figure 2B,D illustrate the expansion of the detected hyper and hypo DMRs’ annotation, respectively. To clarify, the majority of the DMRs located in the intergenic regions were expanded to the intronic regions both in hyper and hypo categories (Fig. 2).

We assessed the distribution of DNA methylation changes across the genomic features based on fold difference score in hyper- and hypo-methylation (Supplementary Fig. S1). The distribution of DNA methylation changes in promoter (opensea) and gene body (opensea) were lower than other features in hyper-methylation, while promoter (island, shore, shelf) and gene body (island, shore, shelf) had similar variation. The distribution of DNA methylation changes in promoter (opensea) and gene body (island, shore, shelf and opensea) were lower than promoter (island, shore, shelf) in the hypo-methylation state. The variation of fold difference score in hyper- and hypo-methylation ranged from 0.2 to 6.6 and 0.3 to 6.3, respectively.

**Figure 3:**
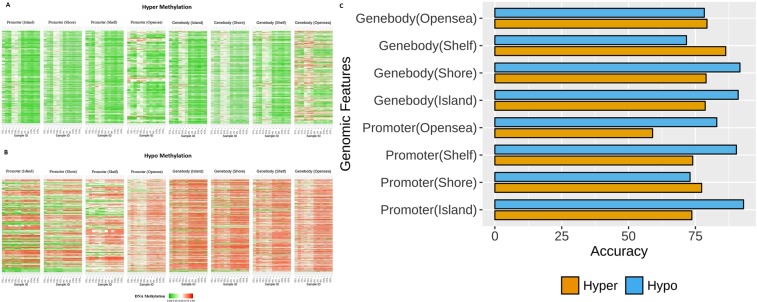
Heatmap representation of Human Methyl-seq data between CRC and normal samples in (**A**) hyper- methylation (**B**) hypo- methylation DMRs on different genomic features [Promoter (Island), Promoter (Shore), Promoter (Shelf), Promoter (Opensea), Genebody (Island), Genebody (Shore), Genebody (Shelf),Genebody (Opensea)], (**C**) Accuracy of SVM classification model on different subsets of genes.

In the present study, we evaluated the overall DNA methylation patterns across different genomic features in hyper- and hypo-methylation states (Fig. 3A,B). We assessed the prediction performance with support vector machine classification method (SVM)^20^ towards the discrimination between CRC and normal groups on DNA methylation patterns from different genomic features. The most accuracy predictors were the gene body (shelf) 86.33% followed by the gene body (opensea) 79.33%, whereas the promoter (opensea) was 54% that is the least accuracy in hyper-methylation patterns. In hypo-methylation patterns, promoter (island) and gene body (shore) had the most accuracy predictors in the values of 93% and 91.66%, respectively. However, the gene body (shelf) was 71.66%, which is the least accuracy in hypo-methylation patterns. The accuracy of SVM^20^ classification on DNA methylation patterns across different genomic features were shown in (Fig. 3C).

**Figure 4:**
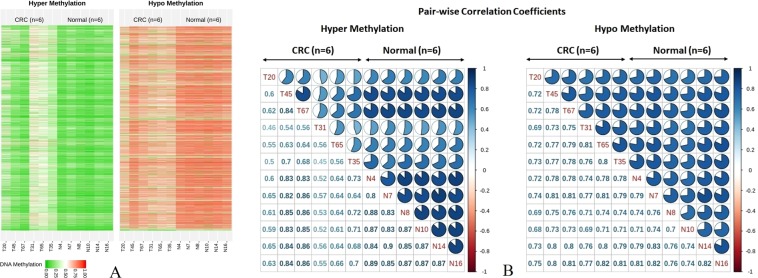
(**A**) Heatmap representation of DNA Human Methyl-seq data between CRC and normal samples on whole genes. Each column represents one sample and each row represents CpGs methylation status in hyper- hypo Methylation DMRs identified by DMRFusion. (**B**) Pair-wise correlation coefficients matrix comparing DNA methylation between CRC and normal groups in hyper- hypo methylation.

We performed comprehensive DNA methylation profiling based on the detected hyper and hypo DMRs between our CRC and normal groups, illustrated in Fig. 4A. We also assessed the mutation status of *KRAS* and *BRAF* in all samples. Three out of 6 CRC samples (T31, T35 and T65) were detected as positive- *KRAS* somatic mutation but no mutation was detected in normal samples. *BRAF* somatic mutation was detected in only T20. A hierarchical model (hCluster) clustering approach was used on the 315606 variable CpGs. We identified 3 distinct CRC subgroups, described as cluster 1 (N = 1, T20), cluster 2 (N = 2, T45 & T67) and cluster 3 (N = 3, T31, T65 & T35), having mutation status of positive *BRAF* and negative *KRAS* in cluster 1, negative *BRAF* and negative *KRAS* in cluster 2 and negative *BRAF* and positive *KRAS* in cluster 3. Cluster 1 and cluster 3 were represented as intermediate- and high-relative methylation among CRC samples, while cluster 2 with low-relative methylation, was similar to the normal relative methylation pattern. DNA methylation changes’ pattern among CRC samples in hyper- and hypo-methylation were higher and lower than normal samples, respectively. Figure 4B shows a pair-wise correlation coefficients of DNA methylation changes’ pattern in hyper- and hypo-methylation between CRC and normal samples. The overall CpG methylation profiles between cluster 2 (T45 & T65) and normal samples were highly correlated as pairwise correlation coefficients ranged from 0.80 to 0.89 in hyper-methylation, while the correlation between cluster 1 and cluster 3 were low, ranged between 0.4 and 0.5 in hyper-methylation. Furthermore, the correlation between CRC and normal samples were ranged from 0.6 to 0.8 in hypo-methylation.

#### Robust genes in CRC and gene ontology

**Figure 5:**
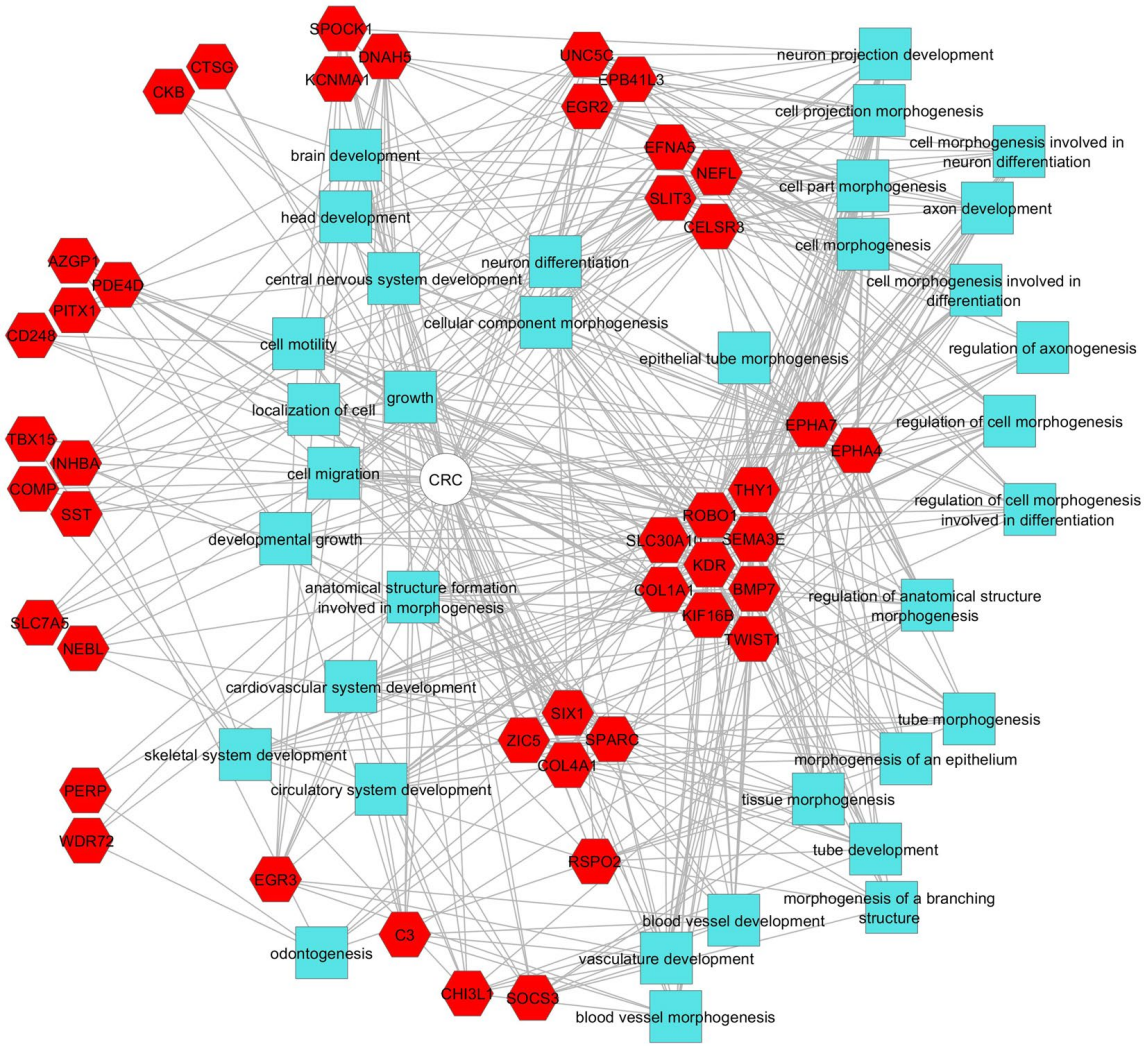
Functional enrichment analysis of regulatory network of hyper/hypo methylated genes with significant changes in transcription level.

Online analysis was performed by GEO2R software to identify DEGs (differentially expressed genes) or DMGs (differentially methylated genes). By comparing DEGs from the expression datasets with the current experiments, we detected 14 common DEGs including 4 hyper-methylated/down-regulated, 2 hyper-methylated/up-regulated, 4 hypo- methylated/down-regulated and 4 hypo-methylated/up-regulated. The detailed information regarding expression levels were shown in Supplementary Table S2. Common DMGs between the current experiments and the methylation datasets were 827 genes in 2 categories including 449 hyper-methylated genes and 378 hypo-methylated genes. According to the flowchart that was presented in Fig. 1, by comparing these differentially methylated genes with gene expression datasets from microarray datasets, 78 common genes were recognized as functionally methylated genes. Gene ontology of these genes demonstrated that the majority of terms were related to cell differentiation, tissue development and embryonic organ morphogenesis (Fig. 5 and Supplementary Fig. S2).

Besides, comparing these 78 genes with methylation arrays resulted in only 3 common genes *SLC30A10* (hyper- methylation/down-regulated), *INHBA* (hypo-methylation/up-regulated) and *CLDN1* (hypo-methylation/up-regulated) that were robustly appeared among previous studies and our current experiment. Statistics information from expression and methylation values for candidate genes were shown in Table 1.

**Table 1:** Statistical information of expression and methylation values in candidate genes (*SLC30A10*, *CLDN1* and *INHBA)*.

Group	Gene Abbreviation	Gene Full Name	Current experiment (FDS)	Expression array (logFC)			
				GSE28000	GSE21815	GSE44076	GSE68468
Hyper Methylation Down regulated	*SLC30A10*	Solute Carrier Family 30 Member 10	0.37	−2.04	−2.87	−2.16	−2.38
				**Methylation array (logFC)**			
				**GSE48684**	**GSE53051**	**GSE77718**	**GSE101764**
				0.28	0.44	0.25	0.19
Hypo Methylation Up regulated	*CLDN1*	Claudin 1	2.29	**Expression array (logFC)**			
				**GSE28000**	**GSE21815**	**GSE44076**	**GSE68468**
				2.29	3.14	5.09	4.98
				**Methylation array (logFC)**			
				**GSE48684**	**GSE53051**	**GSE77718**	**GSE101764**
				−0.17	−0.38	−0.22	−0.31
Hypo Methylation Up regulated	*INHBA*	Inhibin beta A subunit	1.54	**Expression array (logFC)**			
				**GSE28000**	**GSE21815**	**GSE44076**	**GSE68468**
				2.88	4.64	3.51	5.12
				**Methylation array (logFC)**			
				**GSE48684**	**GSE53051**	**GSE77718**	**GSE101764**
				−0.168	−0.24	−0.21	−0.23

#### Technical and biological validations

The technical validation was performed by methylation sensitive high-resolution melting (MS-HRM) on the discovery sample set in CRC and normal control groups. They were accordingly discriminated based on methylation sequencing results.

Methylated DNA markers (MDMs) were identified for our candidate genes and primers and probes were designed and synthesized for the top MDM considered as a diagnostic biomarker.

The candidate MDMs, hyper- methylated/down-regulated, were tested by MethyLight assay on DNA extracted from 39 and 47 formalin-fixed paraffin-embedded (FFPE) case and control tissues, respectively. We also performed the experiment on 28 case and 26 control fresh tissues. The results showed that the methylation at MDM was significantly different (*t*-test, p-values < 2.2e-16; mean of Cq case 29.58 and mean of Cq control 36.27 for FFPE tissues and *t*-test, p-values = 1.163e-07; mean of Cq case 29.69 and mean of Cq control 34.96 for fresh tissues) with the sensitivity of 87.17% and 82.14% at a specificity cut-off of 100% and 92% for FFPE and fresh tissues, respectively (Table 2).

**Table 2:** Patients and the tumor characteristics of CRC adenocarcinoma in FFPE, fresh tissue and plasma studies. *N/A: Not applicable.

Type	Biological FFPE tissue validation		Biological fresh tissue validation		Tissue expression		Plasma study	
	Cases	Control	Cases	Control	Cases	Control	Cases	Control
**Number**	(N = 39)	(N = 47)	(N = 28)	(N = 26)	(N = 33)	(N = 35)	(N = 22)	(N = 20)
**Gender**								
Male	23 (59%)	27 (57%)	19 (68%)	18 (69)	19 (57%)	17 (48%)	13 (59%)	11(55%)
Female	16 (41%)	20 (43%)	9 (32%)	8 (31)	14 (43)	18 (52%)	9 (41%)	9 (45%)
**Age at Diagnosis (Year)**								
Median	58	61	67	59	59	56	67	56
Range	[24–85]	[24–85]	[44–82]	[45–84]	[24–85]	[35–77]	[44–82]	[45–84]
**Tumor site**								
Anal	0	0	0	0	0	0	0	—
Rectum	3 (8%)	4 (8%)	10 (36%)	9 (35%)	2 (6%)	0	8 (36%)	—
Sigmoid	20(51%)	24 (51%)	6 (21%)	12 (46%)	18 (54%)	21 (60%)	4 (18%)	—
Transverse colon	0	0	0	0	0	0	0	—
Descending colon	4 (10%)	5 (11%)	1 (3%)	0	3 (9%)	7 (20%)	1 (5%)	—
Ascending colon	7 (18%)	7 (15%)	3 (11%)	1 (4%)	5 (15%)	0	3 (14%)	—
Cecum	2 (5%)	2 (4%)	8 (29%)	4 (15%)	2 (6%)	0	6 (27%)	—
Entire colon	3 (8%)	5 (11%)	0	0	3 (9%)	7 (20%)	0	—
**TNM stage**								
I	7 (18%)	—	N/A	—	6 (18%)	—	N/A	—
IIA	8 (21%)	—	N/A	—	6 (18%)	—	N/A	—
IIB	2 (5%)	—	N/A	—	2 (6%)	—	N/A	—
IIC	1 (2%)	—	N/A	—	1 (3%)	—	N/A	—
IIIA	2 (5%)	—	N/A	—	2 (6%)	—	N/A	—
IIIB	10 (26%)	—	N/A	—	7 (21%)	—	N/A	—
IIIC	9 (23%)	—	N/A	—	9 (27%)	—	N/A	—
IVA	0	—	N/A	—	0	—	N/A	—
IVB	0	—	N/A	—	0	—	N/A	—
**Tumor size**								
≥4	15 (39%)	—	N/A	—	10 (30%)	—	N/A	—
4.1–7.9	20 (51%)	—	N/A	—	20 (61%)	—	N/A	—
8–11.9	2 (5%)	—	N/A	—	1 (3%)	—	N/A	—
≤12 cm	2 (5%)	—	N/A	—	2 (6%)	—	N/A	—
Mean of Cq	29.7	35.5	29.7	35	32.5	27.8	36.3	0
*KRAS* mutation	19 (49%)	—	9 (32%)	—	12 (36%)	—	6 (27%)	—
*BRAF* mutation	9 (23%)	—	3 (11%)	—	5 (15%)	—	2 (9%)	—
**Diagnostic**								
Sensitivity	87%		82%		—		41%	
Specificity	100%		92%		—		100%	

To evaluate the ability of the entire biomarker to correctly classify between the two possible conditions (cancerous vs. control), we built a support vector machine learning model (SVM)^20^, using the GSE42752 dataset as a training set and the GSE52270 as a test set with 10-fold cross validation. The sensitivity and specificity results of classification methods were 91.93% and 82.85%, respectively. In total, 329 DMRs were recognized with an impact on transcription.

#### Tissue expression of candidate genes

We performed RT-qPCR to compare the expression profile of candidate gene (*SLC30A10*) between case and control FFPE tissues. The reference gene of *GAPDH* was used as a reference control in the experiment. The expression pattern revealed that the candidate gene was expressed in both case and control FFPE tissues with a significant difference. As shown in Supplementary Fig. S3, normal tissue mRNA elucidated a significant 22.1-fold upregulation as compared to the CRC tissues (p-value < 2.2e-16 mean of Cq case 32.51 and mean of Cq control 27.84) (Table 2). Hence, the expression level was significantly lower in cancerous tissues.

#### Tissue-specific methylation study

In order to study the specificity of the candidate MDM (hyper- methylated/down-regulated), it was also tested by MethyLight assay on gastric, liver and esophagus cancer tissues. The results were statistically not significant (p value > 0/05) indicating that the levels of methylation in these cancers were similar to the normal colon tissue.

#### Plasma pilot study

Methylation of the top MDM was assessed in circulating DNA of CRC patients and normal individuals. Nine out of 22 CRC samples and 20 out of 20 normal samples were identified correctly (Table 2). Besides, the methylation pattern of the top MDM was studied in BLUEPRINT Epigenome database. Based on the BLUEPRINT Epigenome results, the methylation percentage of blood-borne cells for the MDM was lower than 0.10%, indicating a poorly methylation level of hematopoietic cells. It is worth mentioning that the methylation profile of normal blood was similar to the normal colon (data not shown).

## Discussion

DNA CpG methylation is usually associated with a closed state of chromatin and has been widely accepted as an important mechanism to maintain gene repression^21^. In addition to control gene expression, it is known to be a cancer driver mechanism. DNA methylation is negatively correlated with gene expression but so far this association can only be detected for hundreds of genes, and the correlation direction is both positive and negative^22^.

In the current study, we investigated the interplay between CpG methylation and gene expression. Common DMGs between the current experiments and the methylation datasets were 827 genes in 2 categories including 449 hyper-methylated genes and 378 hypo-methylated genes. Gene ontology analysis for methylated genes showed that the majority of pathways were involved in cell differentiation including smooth muscle cell differentiation (GO: 0051145), positive regulation of animal organ morphogenesis (GO: 0110110), embryonic hind limb morphogenesis (GO: 0035116), and positive regulation of cell junction assembly (GO:1901890). Any aberration in these pathways could result in abnormal cell division or differentiation^23^. Three genes: *SLC30A10*, *CLDN1* and *INHBA* were eventually selected. *SLC30A10* was hyper-methylated and down-regulated, *CLDN1* and *INHBA* were hypo-methylated and up-regulated. Based on the annotation, the DMR were located in CpG island/shore, shore and shore of *SLC30A10*, *CLDN1* and *INHBA*, respectively. These results are in agreement with other reports showing that in cancerous tissue distinct methylation profiles are exhibited more frequently within CpG island and shores^19^.

From the candidate genes, MDMs were tested by MethyLight assay on the DNA extracted from 39 and 47 FFPE case and control tissues, respectively. The results showed that the methylation at MDM was significantly different (p-values < 2.2e-16) with a sensitivity of 87.17% at a specificity cut-off of 100%. Methylation studies on fresh tissues (28 CRC vs 26 control samples), indicated very similar results (p-values = 1.163e-07) with a sensitivity of 82.14% at a specificity cut-off of 92%.

In the current study, significant differences in RNA levels were seen in our candidate gene when comparing cases with controls. We reported a negative correlation in methylation and gene expression in *SLC30A10* gene, which was hyper-methylated and down-regulated in the same tissue samples.

SLCs are the second largest family of membrane proteins in the human genome, which transport a broad spectrum of substrates such as nutrients and drugs. The SLC proteins control key physiological functions, including nutrient uptake, ion transport, and could also function as tumor suppressors^24–26^. The tumor suppressive function of SLCs relates to the inhibition of histone deacetylases (HDAC) and intracellular pH regulation^27^. This superfamily are located in all cellular and intracellular organelle membranes, except the nuclear membrane^28^.

In recent years, increasing attention has been given to the role of plasma membrane transporters such as SLCs in cancer. Some SLCs are upregulated in tumor cells due to higher energy and nutritional requirements^29^.

The SLC30 family is comprised of 10 members. They are involved in transport of zinc (Zn) and manganese (Mn), which are so important in resisting programmed cell death, since Zn has been demonstrated to play a role in a number of cancers^30^ such as prostate cancer^27^. Besides, Mn is an essential metal that is required for various cellular enzymatic activities^31,32^. It may inhibit the acetylation of histone H3 and H4 by increasing and decreasing the activity of HDAC and histone acetyltransferase (HAT), respectively, which eventually causes cell damage and apoptosis^33^. Shangkuan *et al*. identified *SLC30A10* as a candidate gene among the ten recommended candidate genes correlated with CRC using bioinformatics analysis of microarray data from the GEO database^34^. In a recent study from Yagi *et al*. they reported *SLC30A10* as a methylation marker in the CRC. Furthermore, they suggested that methylation epigenotype significantly correlated with *KRAS* and *BRAF* mutation^35^.

The claudin (CLDN) family, consists of at least 24 members, and their expression depends on the cell type. Recent reports have shown that the expression of *CLDN* genes is often altered in cancers. *CLDN1* is a capital component of tight junctions (Tjs), which plays an essential role in tumorigenesis^36^. The role of CLDN in cancer has not been clearly identified, but a recent study suggested that the *CLDN1*-dependent pathway might be involved in the suppression of CRC expression and correlated to tumor invasiveness and prognostic factors^37^.

INHBA (Inhibin β A) is a member of the transforming growth factor β (TGF-β) superfamily. It is able to grossly induce embryonic stem cell differentiation. Notwithstanding, the INHBA’s role in cancer has not been fully explained. Recent studies have shown that *INHBA* gene is overexpressed in cancer^38^ and correlates with cell proliferation and outcomes in several tumors such as lung^39^, gastric^40^, esophageal^41^, and colorectal^38^ cancers. Wildi *et al*. found that activing A, a homodimer of *INHBA*, is also up-regulated in human CRC^42^. However, only a few studies have investigated the epigenetically regulation of this gene in cancer. Seder *et al*. investigated the role of epigenetic regulation of *INHBA* gene expression in esophageal cancer cells. They suggested that overexpression of *INHBA* may promote cell proliferation and may be affected by promoter methylation and histone acetylation^41^. The MDM identified in this study, presenting a specific methylated region in the *SLC30A10* gene, has not previously been reported.

To suggest the candidate MDM as a blood biomarker, we assessed the epigenetic signatures of white blood cells, which compose the largest portion of circulating DNA in plasma^43^. The methylation pattern of the top MDM was studied in BLUEPRINT Epigenome and based on this database, the methylation percentage of blood-borne cells for the MDM was very low (<0.10%), suggesting our candidate MDM as a serum diagnostic biomarker. In addition, in the plasma pilot study we were able to display the methylation pattern of our MDM in plasma cfDNA in CRC patients although the sensitivity was not relatively high. It remains to be determined if technical refinements can improve this issue. Reassessment of the pre-analytical procedures including collecting larger blood volumes, given that the Epi proColon kit requires at least 3.5 ml of plasma for the evaluation of gene methylation^11^ and utilization of more specific collection tubes designed for ctDNA recovery and standardization of blood processing could ameliorate the plasma results. In addition, advanced technology assay implementation including BEAMing (beads emulsion amplification magnetics), digital droplet PCR, and Target Enrichment Long-probe Quantitative Amplified Signal (TELQAS) could progressively enhance the ability to detect aberrant methylated ctDNA among the total cfDNA in a clinical sample^43^. The performance outcome observed in the pilot plasma testing would need to be corroborated in further plasma studies in order to compare its results with available blood tests such as septin 9 blood test. There is only one FDA-approved plasma-based DNA methylation biomarker test for CRC in the market^44^. These days, finding novel biomarkers that are simple, cost-effective, highly specific and sensitive has led to a large clinical interest.

Despite these encouraging early results, there are several potential limitations to the present study. First, patients were enrolled from a limited referral centers. More centers should be involved. Sample sizes especially in plasma phase were not sufficiently large to study the actual performance of the MDM in plasma. Since the main purpose of this study was to find new biomarkers for CRC detection, a plasma pilot study was performed only to demonstrate the eligibility of the candidate gene as a biomarker in plasma. This plasma study was performed in order to evaluate the feasibility, and improve on the study design prior to conducting the entire research project (an ongoing project by the authors’ group). To establish a sensitive approach for solving the technical problems of cfDNA methylation detection in liquid biopsy, we developed and optimized a single tube methylation detection method by selective capture and bisulfite conversion of cfDNA on magnetic beads (unpublished data). This method could significantly improve pre-analytical phase of the diagnostic test leading to a higher sensitivity and specificity for the candidate biomarker in plasma. Thus, further plasma phase studies are needed. The overall sensitivity will be improved with optimized methods as described earlier. Last but not least, further studies are needed to evaluate the organ site specificity of the MDM candidate. In this initial study, we explored a novel MDM for the detection of CRC. There is a clinical feasibility that the candidate MDM could be used for early detection of CRC. Besides, our candidate MDM could be also studied in precancerous lesions, along with the oncogenic cascade from metaplasia through adenoma to CRC.

## Materials and Methods

### Study design

This study had multiple components illustrated in Fig. 1. Following sample collection, it began with a discovery step based on unbiased methylome sequencing using SureSelect^XT^ Methyl-Seq. Regions demonstrating significant differential methylation were identified and technically validated as candidate DMRs. Candidate methylated genes were chosen based on the shared genes with several other methylation (GSE48684, GSE53051, GSE77718, GSE101764) and expression (GSE28000, GSE21815, GSE44076, GSE68468) datasets. MDMs were chosen based on several criteria including methylation fold change, absolute methylation difference, area under the receiver-operator curve and percentage of control sample methylation.

### Biospecimen sources

The current study was approved by Mashhad University of Medical Sciences (MUMS) ethics committee (approval number: 975011) and all methods were performed in accordance with the relevant guidelines and regulations of MUMS. Informed written consent had been obtained from all participants in this study. Patient samples with adenocarcinoma of CRC (N = 6) had a tentative diagnosis determined by colonoscopy. The lesions were removed during endoscopy and confirmed by pathology evaluation at Reza Radiotherapy and Oncology Center (RROC) and Mashhad Pathobiology laboratory by two gastroenterology expert pathologists. Paraffin embedded tissue samples were obtained from Razavi Hospital, Mashhad, Iran. CRC patients with stages I, II & III diseases were included. Excluding criteria of the study were patients with previous CRC, other cancers, positive familial history of adenoma polyposis, inflammatory bowel disease, hereditary CRC and patients with incomplete colonoscopy and documentations. Colorectal tissue controls (N = 6) were taken from individuals who underwent CRC screening by colonoscopy who were negative for adenomatous polyps and CRC through the entire colon and rectum. Demographic characteristics, colonoscopy reports, history of drug, and smoking as well as medical history were collected. The location of lesion was defined as anal, rectum, sigmoid, transverse colon, descending colon, ascending colon, and cecum. The data is presented in Supplementary Table S3.

### Discovery

#### SureSelect^XT^ Methyl-Seq

First, the purity and quantity of DNA tissue samples were evaluated. For passing sample quality control, we included the minimum condition [concentration ≥ 50 ng/*μ*$\ell$, purity (A260/A280) ≥ 1.7, volume ≥ 20 ng/*μ*$\ell$, total amount ≥ 3.0*μ*g]. The global methylation profiles of cancer and normal colon tissues were analyzed using SureSelect^**XT**^ Human Methyl-Seq. This platform assesses 84 mega bases (MB) of genome, 3.7 million CpGs, 19.6 Mb CpG islands, 9.8 Mb cancer- and tissue- specific DMRs, 37 MB GENCODE promoters, 48 MB enhancers, CpG island shores/shelves ± 4 Kb and DNase I hypersensitive sites^45^. Sequencing was performed on the Illumina HiSeq. 4000 (Macrogen Co., South Korea).

#### Identification of DMRs

To detect DMRs, we used the DMRFusion tool which could assess the optimal and significant hyper- and hypo-methylation DMRs in the genome with minimum redundancy and maximum relevance between cancer and control groups. DMRFusion describes the annotation of DMRs such as the nearest transcription start site (TSS), CGIs/shores/shelves, and regions within genome and visualizes DMRs with high fold difference score (p-value and FDR < 0.05 and type I error < 0.01) as described earlier^17^. The Human Methyl-Seq analysis was composed of three steps in the pre-processing stage before detecting DMRs. Firstly, the total reads were assessed by Quality Control (QC) tool^46^ in order to provide informative global and graphical representations of methylation sequencing read quality, prior and after alignment. In this study, our data had high quality in raw sequencing reads among all samples. Secondly, the raw sequencing reads were cleaned by Trim Galore (https://www.bioinformatics.babraham.ac.uk/projects/trim_galore/) to clip sequencing adapters (Illumina universal adapter) and low quality bases (Q < 67 in Illumina) in the 3′ and ambiguous bases in both reads. Thirdly, these raw bisulfite sequencing data were converted into a number of methylated reads and covered reads of cytosines (including unmethylated/methylated reads) by aligning them to the human reference genome (GRCh37/19) using the Bismark tool^47^.

#### Identification of shared differentially regions

In the present study, gene expression profiling datasets (GSE28000, GSE21815, GSE44076, GSE68468) and gene methylation profiling datasets (GSE48684, GSE53051, GSE77718, GSE101764) were obtained from Gene Expression Omnibus (GEO, https://www.ncbi.nlm.nih.gov/geo/), of the National Center for Biotechnology Information (NCBI). Gene expression included data from 509 CRC and 154 normal mucosa tissue samples. Hundred and ninety three CRC and 161 normal mucosa specimens were enrolled in gene methylation profiling datasets. Data from each microarray and methylation were separately analyzed by online software GEO2R (http://www.ncbi.nlm.nih.gov/geo/geo2r/), in order to analyze the DMGs or DEGs by comparing two groups of samples (CRC and normal mucosa tissue) across setup conditions in a GEO series. These DEGs and DMGs were compared with our current experiment results (hypo- or hyper-methylation DMRs) in order to detect robust hypo- or hyper- methylation genes existing in different populations.

In this study, we used p-value and adjusted p-value < 0.05 as the cut-off standard to define DEGs with absolute |fold change| > 2 and DMGs with absolute |fold change| > 0.1. Common hypo-, or hyper- methylation genes were suggested by comparing gene expression and methylation datasets with the result of our current experiment. Thus, the methylation status of the candidate genes were in accordance with gene regulation through gene-specific mechanisms. The overlapping down-regulated and hyper-methylation genes were identified as hyper-methylated, and poorly expressed. Similarly, overlapping upregulated and hypo-methylation genes were considered hypo-methylated, and highly expressed genes. The flowchart illustrating our bioinformatics analysis was presented in Fig. 1.

According to the following criteria: (1) [case/control] methylation fold change (FC) > 20, (2) [case – control] absolute methylation difference (AMD) > 0.10, (3) area under the receiver-operator curve (AUC) > 0.80, p-value < 0.01, (4) control sample methylation < 1.0%, the candidate MDM was identified among the candidate genes derived from Fig. 1.

#### Technical validation

Methyl-Seq data reliability was validated by MS-HRM analysis. The MS-HRM technical validation was performed on the discovery sample set. Briefly, primers specific for bisulfite converted sequences were designed (MethPrime 2.0 software package) and synthesized (Metabion, Germany). Prior to use, MS-HRM assays were evaluated on methylated and un-methylated bisulfite converted control DNA. Optimal annealing temperatures were determined empirically. Genomic DNA isolated from fresh cancerous and normal colon tissue samples were quantitated by Epoch Microplate Spectrophotometer (Winooski, Vermont, USA). Subsequently, DNA was bisulfite-converted using EpiTect Fast Bisulfite Conversion Kit (Qiagen, Germany) according to the manufacturer’s instructions and amplified using the LightCycler 96 (Roche, Mannheim, Germany).

### Biological validation

#### Tissue validation

Specific probe and primers for MethyLight assay were designed (MethPrime 2.0 software package) and synthesized (Bioneer Corporation, South Korea). The MethyLight assay was run on DNA extracted from 39 and 47 independent FFPE and 28 and 26 fresh case and control tissues, respectively. The DNA was extracted using the QiaAmp FFPE Tissue Mini kit (Qiagen, Germany) and QIAamp® Fast DNA Tissue Kit (Qiagen, Germany) for FFPE (5 sections, 5–10 µm thick) and fresh tissues (5–25 mg, each specimen), respectively. They were bisulfite treated with the EpiTect Fast Bisulfite Conversion Kit (Qiagen, Germany) as described above. For MethyLight assay, the multiplex PCR reactions were performed on bisulfite converted DNA using QuantiTect Multiplex PCR (Qiagen, Germany). All amplifications were assayed on the LightCycler 96 (Roche, Mannheim, Germany), and the results were normalized to the β-actin from the same sample.

#### *KRAS* and *BRAF* mutation detection

All samples were tested for the most common and known *KRAS* and *BRAF* mutations for exon 12 and 15, respectively based on a protocol previously published^48,49^. The details are described in Supplementary Fig. S4 and Table 2.

#### Tissue expression of candidate gene

Total RNA from 33 and 35 FFPE case and control tissues were isolated using RNeasy FFPE kit (Qiagen, Germany), according to the manufacturer’s protocol. The RNA was quantified by measuring absorbance at 280, 260 and 230 nm in Epoch Microplate Spectrophotometer (Winooski, Vermont, USA). cDNA was synthesized using the RocketScript RT premix (Bioneer, Korea). The gene specific primer targeting the candidate, *SLC30A10*, and *GAPDH* genes were designed (by primer premier 6 software) and synthesized (Eurofins, Germany). Quantitative real-time RT-PCR reaction was carried out using HOT FIREPol qPCR mix with EvaGreen (Solis BioDyne- Estonia) on the LightCycler 96 (Roche, Mannheim, Germany) and the experiment was conducted in duplicate for each sample. Five point-standard curve was tested for each primer using the pooled cDNA from all samples. The pooled cDNA, serially diluted in nuclease free water by 2 fold, was used as template for real time RT PCR. After the PCR, mRNA levels were normalized to *GAPDH* and the relative expression was determined using the 2-ΔΔCT method. All experiments were carried out according to the digital MIQE guidelines^50^.

#### Tissue-specific methylation study

We also tested the MDM identified in this study (hyper-methylated/down-regulated) in FFPE tissues of other cancers, including gastric (N = 12), liver (N = 7) and esophagus (N = 8) cancers. The DNAs were extracted using the QiaAmp FFPE Tissue Mini kit (Qiagen, Germany) and bisulfite treated with the EpiTect Fast Bisulfite Conversion Kit (Qiagen, Germany). For MethyLight assay, the multiplex PCR reactions were performed as described above.

#### Plasma pilot study

Prior to plasma pilot study, the methylation behavior of the top MDM in blood was explored using online BLUEPRINT tool (Software release: 1.0.5, EnsEMBL version: 79, http://www.blueprint-epigenome.eu/). In the plasma study, the top MDM was assessed with MethyLight assay on cfDNA obtained from CRC patients and normal individuals. The cfDNAs were extracted from 1–3 mL plasma of 22 cases and 20 controls using QIAamp Circulating Nucleic Acid Kit (Qiagen, Germany) according to the manufacturer’s protocol and bisulfite-converted (as above). A multiplex PCR reaction was performed on bisulfite converted DNA for the candidate MDM and *β-actin* gene. The MethyLight assay was normalized to the products of *β-actin* gene. The PCR run was performed in triplicates and similar to the conditions of tissue validation.

#### Gene ontology analysis

Hyper- and hypo-methylation DMRs with a significant impact on transcription were selected to draw a network of GO terms and pathways by ToppCluster^51^ and ClueGo (2.5.3), a Cytoscape application^52^. Both networks were visualized by Cytoscape (version 3.7.1). Molecular Function, Cellular Components and Biological Process terms were selected as standard GO terms in both applications.

## Supplementary information

Supplementary information
