# Supplementary material for: Crosstalk between DNA methylation and gene expression in colorectal cancer, a potential plasma biomarker for tracing this tumor
Source: Sci Rep. 2020 Feb 18;10:2813. doi: 10.1038/s41598-020-59690-0 (PMC7028731; doi:10.1038/s41598-020-59690-0)
Supplement: Supplementary file 1 — Supplementary information [file 41598_2020_59690_MOESM1_ESM.docx]

**Crosstalk between DNA methylation and gene expression in colorectal cancer, a potential plasma biomarker for tracing this tumor**

Mohammad Amin Kerachian^1,2,3^*, Ali Javadmanesh^3,4^, Marjan Azghandi^3, 4^, Afsaneh Mojtabanezhad Shariatpanahi^3^, Maryam Yassi^3^, Ehsan Shams Davodly^3^, Amin Talebi^1,2^, Fatemeh Khadangi^5^, Ghodratollah Soltani^6^, Abdorasool Hayatbakhsh^6^, Kamran Ghaffarzadegan^7^

^1^ Medical Genetics Research Center, Mashhad University of Medical Sciences, Mashhad, Iran.

^2^ Department of Medical Genetics, Faculty of Medicine, Mashhad University of Medical Sciences, Mashhad, Iran

^3^Cancer Genetics Research Unit, Reza Radiotherapy and Oncology Center, Mashhad, Iran

^4^ Department of Animal Science, Faculty of Agriculture, Ferdowsi University of Mashhad, Mashhad, Iran

^5^ Institut Universitaire de Cardiologie et de Pneumologie de Québec, Université Laval, Québec, Québec, Canada

^6^ Department of Gastroenterology, Reza Radiotherapy and Oncology Center, Mashhad, Iran

^7^ Razavi Cancer Research Center, Razavi Hospital, Imam Reza International University, Mashhad, Iran

* Correspondence to: Mohammad Amin Kerachian M.D., Ph.D.

Department of Medical Genetics, Faculty of Medicine, Mashhad University of Medical Sciences, Azadi Square, Mashhad, Iran. Postal code: 917794-8564, Tel: +98 51 38002244

E-mail: [amin.kerachian@mail.mcgill.ca](mailto:amin.kerachian@mail.mcgill.ca) or [kerachianma@mums.ac.ir](mailto:kerachianma@mums.ac.ir)

**Supplementary Figures**


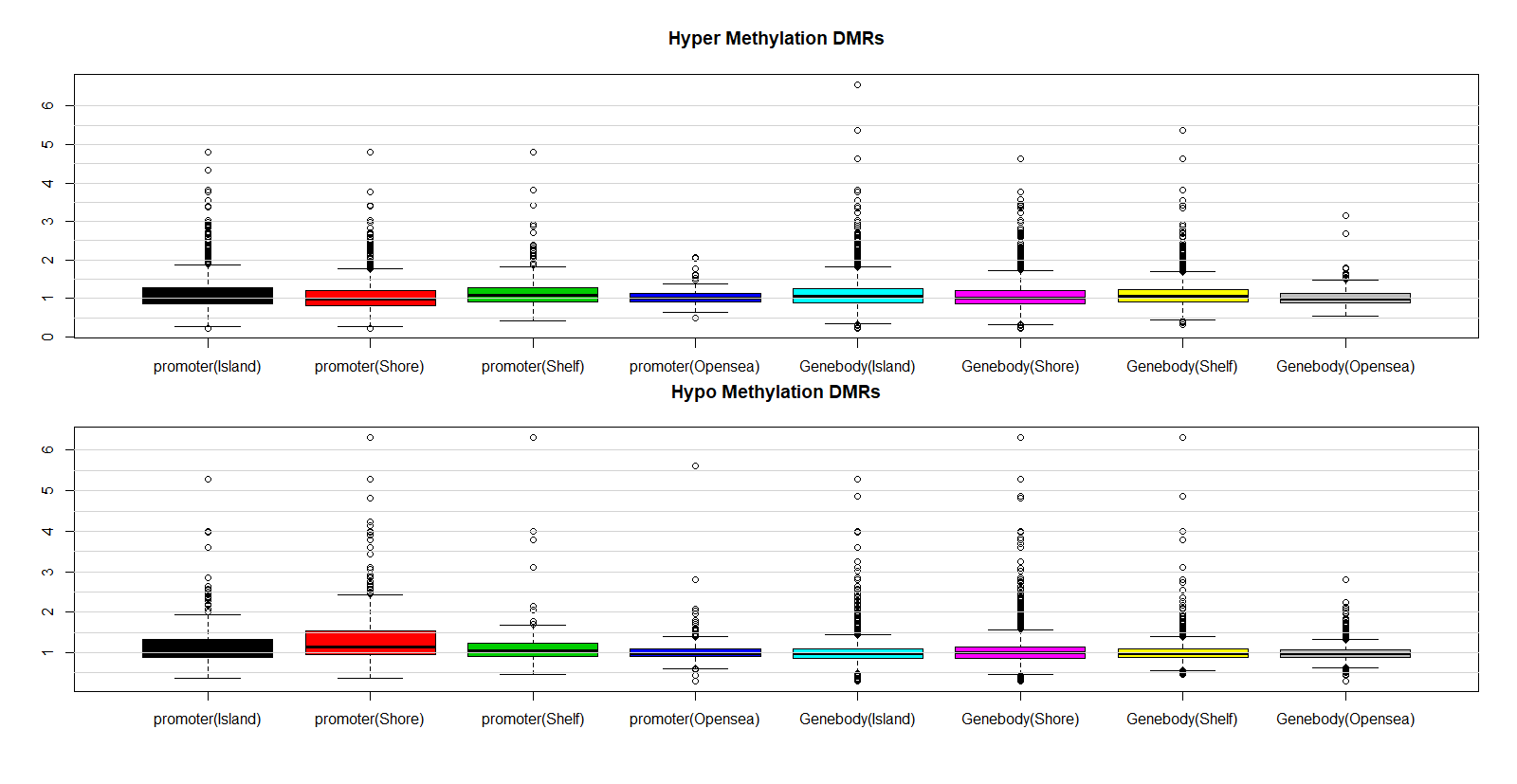


**Figure S1.** Comparison of fold difference score of hypo- or hyper- methylation DMRs identified in different genomic features [Promoter (Island), Promoter (Shore), Promoter (Shelf), Promoter (Opensea), Genebody (Island), Genebody (Shore), Genebody (Shelf) ,Genebody (Opensea)].


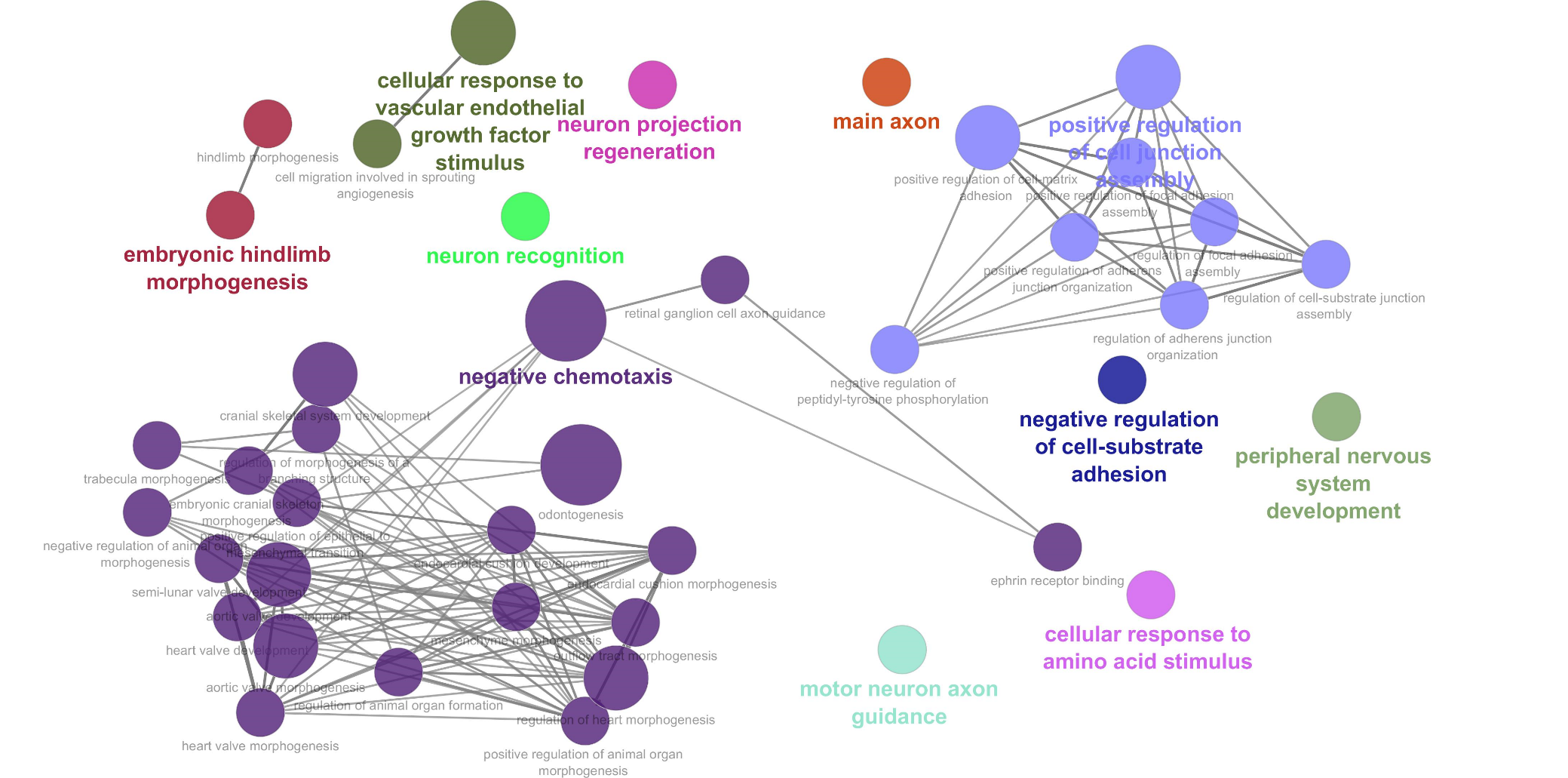


**Figure S2.** Visualization and integration of GO terms and pathways of hyper/hypo methylated genes with significant changes in transcription level by ClueGo.


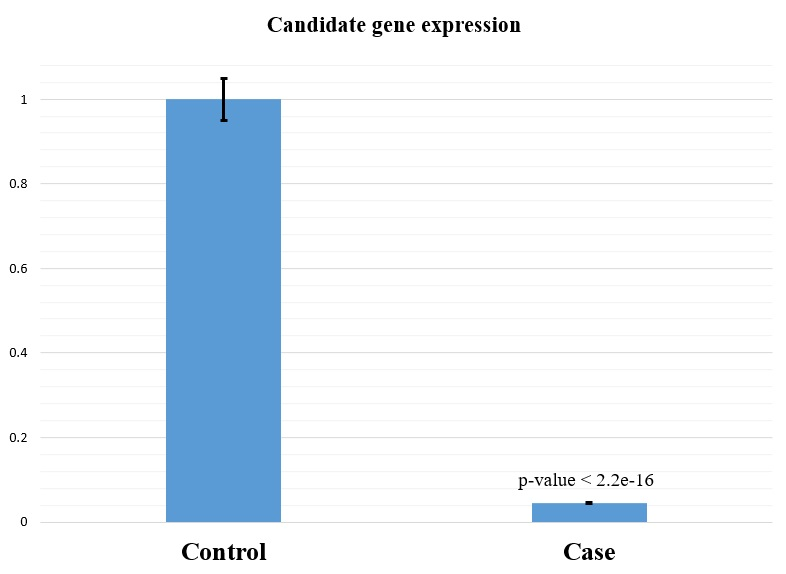


**Figure S3.** Relative expression profile of candidate gene (SLC30A10) in case and control FFPE tissues.


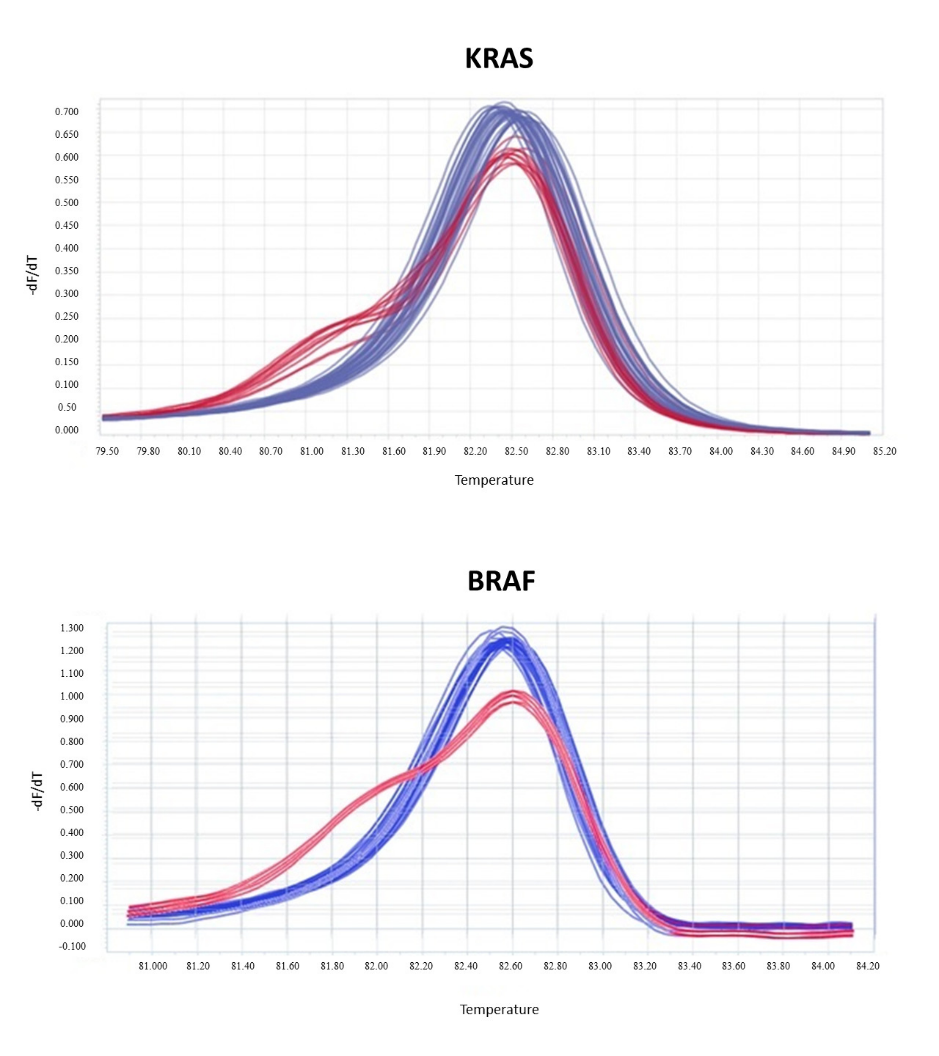


**Figure S4.** High Resolution Melting peaks of KRAS exon 2 (codon 12) and BRAF exon 15 (V600E) analysis, wild-type (blue) and mutant samples (red).

| Table S1. Summary statistics of methylation sequencing data | | | | | | |
| --- | --- | --- | --- | --- | --- | --- |
| Sample | **Total reads** | **Mapping Rate** | **Percentage methylation (CpG context)** | **Average CpG Methylation** | **Average CpG Coverage** | **GC** |
| T65 | 76,723,684 | 88.50% | 47.70% | 0.46 | 24.15 | 27.04% |
| N16 | 70,443,130 | 88.70% | 45.70% | 0.46 | 23.53 | 27.26% |
| T20 | 67,394,464 | 88.90% | 44.70% | 0.44 | 19.58 | 27.03% |
| N4 | 68,165,382 | 88.80% | 46.50% | 0.47 | 22.19 | 27.19% |
| T31 | 61,789,306 | 89.00% | 46.90% | 0.47 | 21.69 | 26.92% |
| N10 | 57,311,634 | 89.05% | 46.70% | 0.47 | 19.26 | 27.04% |
| T35 | 79,004,644 | 88.90% | 46.10% | 0.46 | 24.43 | 27.11% |
| N7 | 75,663,274 | 89.00% | 47.20% | 0.49 | 22.62 | 27.04% |
| T45 | 64,188,480 | 89.00% | 47.40% | 0.47 | 21.22 | 27.06% |
| N8 | 57,091,968 | 89.80% | 46.80% | 0.46 | 20.42 | 27.41% |
| T67 | 61,203,576 | 89.30% | 44.30% | 0.44 | 20.77 | 27.17% |
| N14 | 66,871,860 | 89.60% | 47.40% | 0..47 | 22.17 | 27.11% |

**Supplementary Tables**

| Table S2. Statistical information of 14 common genes in microarray datasets (GSE28000, GSE21815, GSE44076, GSE68468). | | | | | | | |
| --- | --- | --- | --- | --- | --- | --- | --- |
| Group | **Gene Abbreviation** | **Gene Full Name** | **Current experiment**  **(FDS)** | **Expression array (logFC)** | | | |
|  |  |  |  | GSE28000 | GSE21815 | GSE44076 | GSE68468 |
| Hyper methylation Down regulated | *CA I* | Carbonic anhydrase 1 | 1.56 | -3.17 | -7.58 | -7..27 | -4.80 |
|  | *CXCL12* | C-X-C motif chemokine ligand 12 | 1.23 | -3.07 | -2.23 | -2.11 | -3.05 |
|  | *NPY1R* | Neuropeptide Y receptor Y1 | 1.09 | -2.06 | -2.65 | -3.12 | -2.33 |
|  | *SLC30A10* | Solute Carrier Family 30 Member 10 | 0.37 | -2.04 | -2.87 | -2.16 | -2.38 |
| Hyper methylation Up regulated | *ASCL2* | Achaete-Scute Family BHLH Transcription Factor 2 | 1.92 | 2.39 | 4.04 | 3.67 | 2.89 |
|  | *KLK10* | Kallikrein Related Peptidase 10 | 1.1 | 2.40 | 5.48 | 2.05 | 3.14 |
| Hypo methylation Down regulated | FCGBP | Fc Fragment Of IgG Binding Protein | 1.22 | -2.33 | -4.36 | -3.90 | -4.15 |
|  | *ANPEP* | Alanyl Aminopeptidase, Membrane | 1.18 | -2.37 | -2.98 | -5.53 | -2.87 |
|  | *PLAC8* | Placenta Specific 8 | 1 | -2.08 | -2.17 | -4.58 | -2.37 |
|  | *GUCA2A* | Guanylate cyclase activator 2A | 0.74 | -2.19 | -5.25 | -6.32 | -4.52 |
| Hypo methylation Up regulated | *CLDN1* | Claudin 1 | 2.29 | 3.14 | 5.09 | 4.98 | 2.13 |
|  | *INHBA* | Inhibin beta A subunit | 1.54 | 2.88 | 4.64 | 3.51 | 5.12 |
|  | *MMP1* | Matrix metallopeptidase 1 | 1.09 | 3.66 | 2.67 | 3.83 | 3.01 |
|  | *TACSTD2* | Tumor-Associated Calcium Signal Transducer 2 | 0.97 | 2.16 | 3.43 | 3.08 | 2.11 |

| Table S3. Patient characteristics in CRC and normal individuals | | | | | | | | | |
| --- | --- | --- | --- | --- | --- | --- | --- | --- | --- |
| CRC patients | | | | | | | | | |
| ID | **Age** | **Sex** | **Drug** | **Smoking** | **History of colon disease** | **Personal history of cancer (Year)** | **Family history of cancer -Relative (Age)** | **Tumor location** | **Pathology result** |
| 65T | 56 | MALE | YES | YES | NO | NO | NO | Cecum | Well differentiated Adenocarcinoma |
| 20T | 59 | MALE | NO | NO | NO | NO | NO | Cecum | Adenocarcinoma, moderately differentiated |
| 31T | 61 | MALE | YES | YES | NO | NO | Leukemia-Second relative(37) | Sigmoid | Adenocarcinoma, moderately differentiated |
| 35T | 71 | MALE | NO | NO | NO | NO | Breast- Sister(40)-child(35) | Rectum | Adenocarcinoma, moderately differentiated |
| 45T | 69 | MALE | NO | NO | NO | NO | Liver-Brother(80),  second relative(50) | Rectum | Adenocarcinoma, moderately differentiated |
| 67T | 70 | MALE | NO | NO | NO | NO | NO | Rectosigmoid | Adenocarcinoma, well differentiated |
| Normal individuals | | | | | | | | | |
| ID | **Age** | **Sex** | **Drug** | **Smoking** | **History of colon disease** | **Personal history of cancer (Year)** | **Family history of cancer -Relative (Age)** | **Biopsy location** | **History of disease** |
| 16N | 60 | MALE | YES | NO | NO | NO | Brain-Sister(56) | Cecum | Diabetes, Anemia, Liver/Heart/Kidney diseases |
| 4N | 56 | MALE | NO | NO | NO | NO | NO | Cecum | Anemia |
| 10N | 60 | MALE | YES | YES | NO | NO | NO | Sigmoid | Heart disease |
| 7N | 74 | MALE | NO | NO | NO | NO | NO | Rectum | Gastrointestinal disease, High blood pressure |
| 8N | 60 | MALE | NO | NO | NO | NO | NO | Rectum | NO |
| 14N | 76 | MALE | NO | NO | NO | NO | NO | Rectosigmoid | NO |
